# Rapid identification of wood species using XRF and neural network machine learning

**DOI:** 10.1038/s41598-021-96850-2

**Published:** 2021-09-02

**Authors:** Aaron N. Shugar, B. Lee Drake, Greg Kelley

**Affiliations:** 1 Garman Art Conservation Department, Buffalo State College – SUNY, Buffalo, NY USA; 2 Department of Anthropology, University of New Mexico, Albuquerque, NM USA; 3 Collections, Curatorial and Conservation Branch, Indigenous Affairs and Cultural Heritage Directorate, Parks Canada, Government of Canada, Ottawa, ON Canada

**Keywords:** Characterization and analytical techniques, Characterization and analytical techniques

## Abstract

An innovative approach for the rapid identification of wood species is presented. By combining X-ray fluorescence spectrometry with convolutional neural network machine learning, 48 different wood specimens were clearly differentiated and identified with a 99% accuracy. Wood species identification is imperative to assess illegally logged and transported lumber. Alternative options for identification can be time consuming and require some level of sampling. This non-invasive technique offers a viable, cost-effective alternative to rapidly and accurately identify timber in efforts to support environmental protection laws and regulations.

## Introduction

The identification of wood species is difficult at best. Typically, analytical techniques that have been used for identification are invasive, require some level of sampling, and can have expenses associated with them^1^. In some cases, the analysis can be undertaken using microscopic techniques^2^, while more advanced techniques using visual learning, chemical analysis and genetics are making rapid strides^3–6^. The importance of being able to identify tree species is essential in the continuing fight to control the illegal trade of lumber^1,7^.

Illegal logging and the trade in illegal timbers are a largely recognized threat to global biodiversity^8,9^. Resolution 23/1 was adopted by the United Nations in May 2014 promoting the development of tools and technologies that could be used in the fight against illicit trafficking of timbers^10,11^. Treaties such as the Convention on International Trade in Endangered Species of Wild Fauna and Flora (CITES) along with current laws and certification schemes provide a framework to protect over-exploited species and control illegal trade^8^. The use of a rapid, non-destructive forensic verification technique to ensure the compliance of wood products as they travel through the entire chain of providers to users would enhance governmental and certification bodies’ efforts^10^. Without species validation, the economic incentive to provide false information is substantial, and timber is easily misattributed to circumvent trade regulations^4^.

Environmental protection laws exemplified by the US Lacey Act require the declaration of full scientific names with genus and species^10^. Since logs and sawn timber lack the morphological characters of leaves, fruits, seeds and flowers traditionally used in species determination^12^, accurate anatomical identification down to the species level both rapidly in the field and through more invasive and time-consuming laboratory investigations can be challenging^1^. This is especially true in tropical forests with high species diversity where the anatomy of a specimen is often not well known^13^.

Traditional microscopic examination of wood sections has been a staple technique to narrow down genus, and in some cases species, but is time consuming and requires specialist knowledge^10^. Front-line officers who perform initial inspections are trained and knowledgeable about related issues, but may have limited or insufficient taxonomical expertise to verify species and make quick decisions as to whether or not the wood in question needs additional scientific testing^14^. While it is recognized that rapid-field identification is important, the cost and effort has often been cited as a hindrance to the process^1^. To overcome the lack of trained anatomists and the analytical limitations for determining species, several separate scientific disciplines have focused their attention on this problem^1,6^.

No one scientific methodology can address all timber identification questions related to genus, species, geographic region of origin, age, and individual species, so multiple approaches are often needed^8^. For the reliable identification of select species, visual learning, chemical analysis, and genetics have been employed^8^. Examples include machine vision^15^, near infrared spectroscopy (NIRS)^12^, direct analysis in real time time-of-flight mass spectrometry (DART TOFMS)^3^, and DNA barcoding^16^. Most of these techniques are in their early stages of development with limited application, but with recognized potential to develop^1^. These methodologies are mainly lab-based systems that are currently expensive and not readily available. They require specific training both to use and interpret resulting data. DNA barcoding relies on extensive databases, and all but NIRS require small samples to be taken for analysis.

The development of a non-destructive, easy to employ, cost effective identifier that can be used by front-line workers would help to improve rapid field-level inspections as well as forensic wood identification. In addition, effective scientific verification at the beginning of the supply chain could have great impact tracing and verifying legal timber along the global supply network in efforts to curb illegal logging and prevent lumber substitution. Such technology could be used as an early screening process or a ‘triage’ that could further separate out tree species and select key trees for additional confirmation testing^1,13,14^. These requirements may well be met by the use of X-ray fluorescence spectrometers which could become a vital tool in the fight against illegally traded lumber and as well as assistance for species identification for other areas of study.

X-ray fluorescence (XRF) is a rapid elemental identifier that has been employed extensively in various fields for the characterization of materials. It has had limited use for the identification of wood mainly because of the slight variations in wood chemistry making it difficult to create accurate calibrations which could allow for the clear separation of genus or species. XRF has successfully been used to identify heavy metals in woods^17^ and has shown some limited use to characterize variable density in wood^18^. This method has recently been taken one step further through the application of X-ray computed tomography, which is being used for wood identification but still requires samples to be taken and is time consuming^19^. The real value in XRF is that it can be portable (portable or handheld XRF), analysis is rapid, and testing can be done on site (scans can often be done in less than one minute per sample). Instrumentation is relatively cheap, has a small footprint, and is easy to learn how to use.

With the advent of more powerful computers and the development of machine learning and artificial intelligence, we are now able to reassess the potential for XRF to perform rapid wood-species identification. XRF analysis combined with machine learning and deep learning neural networks algorithms allow for more detailed investigations of materials that in the past were not seen as possible. For example, by using random forest regression on XRF data, moisture concentrations in manure have been determined^20^. In addition, using neural networks models in conjunction with XRF analysis, the total organic carbon content of geological shale has been analyzed^21^. The application of neural networks, which allows for the continual building of model design, has been fundamental to the development of wood-species identification using XRF as presented here.

The field of artificial intelligence has seen extraordinary progress since the groundbreaking AlexNet model made 2D convolutional neural networks (CNNs) mainstream^22^. CNN models are designed to mirror how vision systems work in animals through the use of shifting positions^23^. These models tend to diverge from other statistical methods in the following ways:

- They can interpret data in context-specific situations (e.g. facial features or the ability to classify and separate paintings by different artists^24^).
- When completed, they can be retrained on new data.

The output of these models is a set of interconnected neural weights that can be retrained as new data is made available. These ongoing ‘learning models’ develop and improve over time. The danger to these models is that they learn too much from datasets, a problem known as overfitting. One potential resolution to this problem is to force neurons to be dropped randomly^25,26^.

CNNs can be 1 dimensional and suited for strings of numbers, not unlike the way XRF spectra are presented. Because of lower dimensionality, these networks require exponentially fewer computer resources to train, making them practical on most modern computer systems. They are particularly well suited for time-series data-classification tasks^27^. These models have been used successfully with spectral data to detect atrial fibrillation^28^ and identify acids in IR spectra^29^, among other applications.

The application of CNN for the identification of wood species has been developing over the last few years. The implementation of CNN combined with visual assessment (imaging) of gross features has been used for the identification of veneers, fine-grained wood specimens, and several tropical wood species^14,30–32^. Images of microscopic sections have also been classified using CNN^33^, and the sorting of several Pinus species has been successfully undertaken using an artificial neural network^34^. To date, there has been no use of XRF in combination with CNN for the identification of wood species.

This paper will present the first use of XRF spectral collection in combination with convolutional neural network machine learning to accurately identify various wood species. As a pilot study, it provides strong evidence for the validity of this rapid, non-destructive identification technique and allows future researchers a path forward for its development and implementation.

## Methodology

### Selection of samples

**Figure 1:**
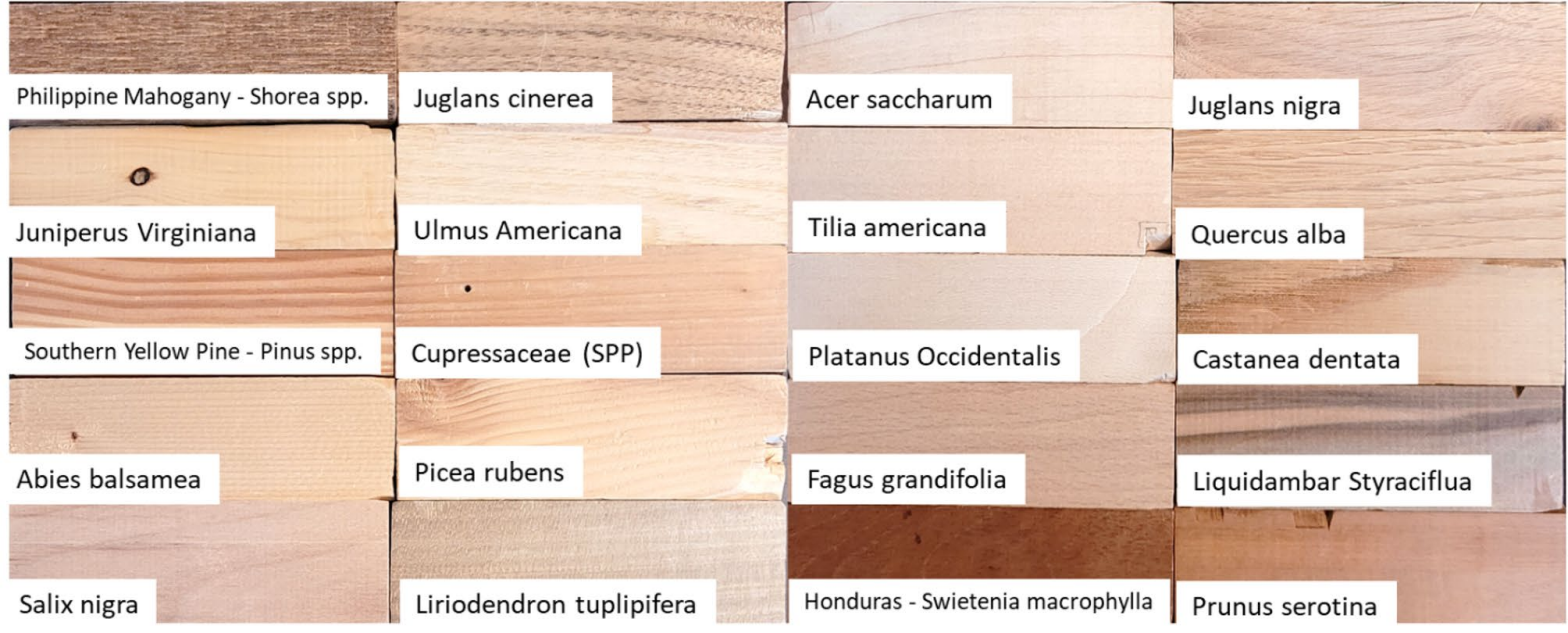
Example of some of the wood samples used in this study (all housed in the Garman Art Conservation Department, SUNY – Buffalo State).

Wood blanks used for this study are housed in the Garman Art Conservation Department, SUNY—Buffalo State. They are part of various educational wood identification sets that were purchased over 20 years ago and consist mostly of heartwood. No samples violated CITES regulations, nor crossed state or international borders during this study (Fig. 1, Table 1). If multiple samples were available of one species, they were used to develop a more robust model. Forty-eight wood specimens were investigated with 17 of those having more than one sample run for comparison giving a total of 66 datasets. Samples were all run on either tangential or radial sections, or slight rotations between these two orientations.

**Table 1:** List of wood species analyzed and the number of samples run for each.

Scientific name	Common names	Samples run
*Abies balsamea*	Balsam fir	1
*Acer saccharum*	Sugar maple	2
*Castanea dentate*	American chestnut	1
*Cedrela odorata*	Spanish cedar	1
*Cedrela* spp.	Cedro	1
*Dyera costulata*	Jelutong	1
*Fagus grandifolia*	America beech	2
*Gleditsia triacanthos*	Honey locust	2
*Juglans cinerea*	Butternut	2
*Juglans nigra*	Black walnut	2
*Juniperus virginiana*	Red cedar	1
*Lagarostrobos franklinii*	Huon pine	1
*Liquidambar styraciflua*	Sweetgum	2
*Liriodendron tulipifera*	Yellow poplar, tulip poplar	2
*Maclura pomifera*	Osage orange	1
*Morus alba*	White mulberry	1
*Morus rubra*	Red mulberry	1
*Ostrya virginiana*	American hophornbeam, ironwood	1
*Picea rubens*	Eastern spruce	1
*Picea rubens*	Red spruce	1
*Picea* spp.	Spruce	1
*Pinus echinata*	Shortleaf pine	1
*Pinus monticola*	Western white pine	1
*Pinus palustris*	Longleaf pine	1
*Pinus ponderosa*	Ponderosa pine, western yellow pine	1
*Pinus* spp.	Southern yellow pine	2
*Pinus strobus*	Eastern white pine	2
*Pinus virginiana*	Virginia pine	1
*Platanus occidentalis*	American sycamore	2
*Populus balsamifera*	Balsam poplar	1
*Populus* spp.	Aspen	1
*Prosopis* spp.	Mesquite	1
*Prunus serotina*	Black cherry	2
*Prunus serrula*	Birch bark cherry	1
*Pyrus communis*	Pearwood	1 ara>
*Quercus alba*	White oak	2
*Quercus falcata*	Southern red oak	1
*Quercus rubra*	Northern red oak	1
*Salix nigra*	Black willow	2
*Sassafras* spp.	Sassafras	1
*Shorea* spp.	Philippine mahogany	2
*Swietenia macrophylla*	Caoba	1
*Swietenia macrophylla*	Honduran mahogany	2
*Taxodium* spp.	Cypress	2
*Thespesia grandiflora*	Maga	1
*Thuja plicata*	Western red cedar	1
*Tilia americana*	Basswood	3
*Ulmus americana*	American elm	1

### Collection of spectra

Blank wood samples were analyzed using a Bruker Tracer 5 g XRF coupled to a DeWitt MSS-150E scanning stage. The XRF was run at 50 kV 35uA with an 8 mm collimator and no filter for 20 s per scan. Samples were placed so that the head of the instrument was no more than 1 mm from the surface of the wood. The scanning stage was set to move at 3 mm in the X axis per scan and then shift up 3 mm in the Y axis to create a matrix that produced approximately 260 scans per wood sample. Proprietary software outputted data as individual Bruker PDZ files. The spectra were then converted to csv files for processing.

### Application of machine learning algorithm

Spectra data were imported using the open-source CloudCal software^35^ with spectra data normalized to total counts (e.g., each detector channel representing a % of the total, rather than counts). The spectrum was then compressed to 0.7 and 37 keV in 100 eV increments.

**Figure 2:**
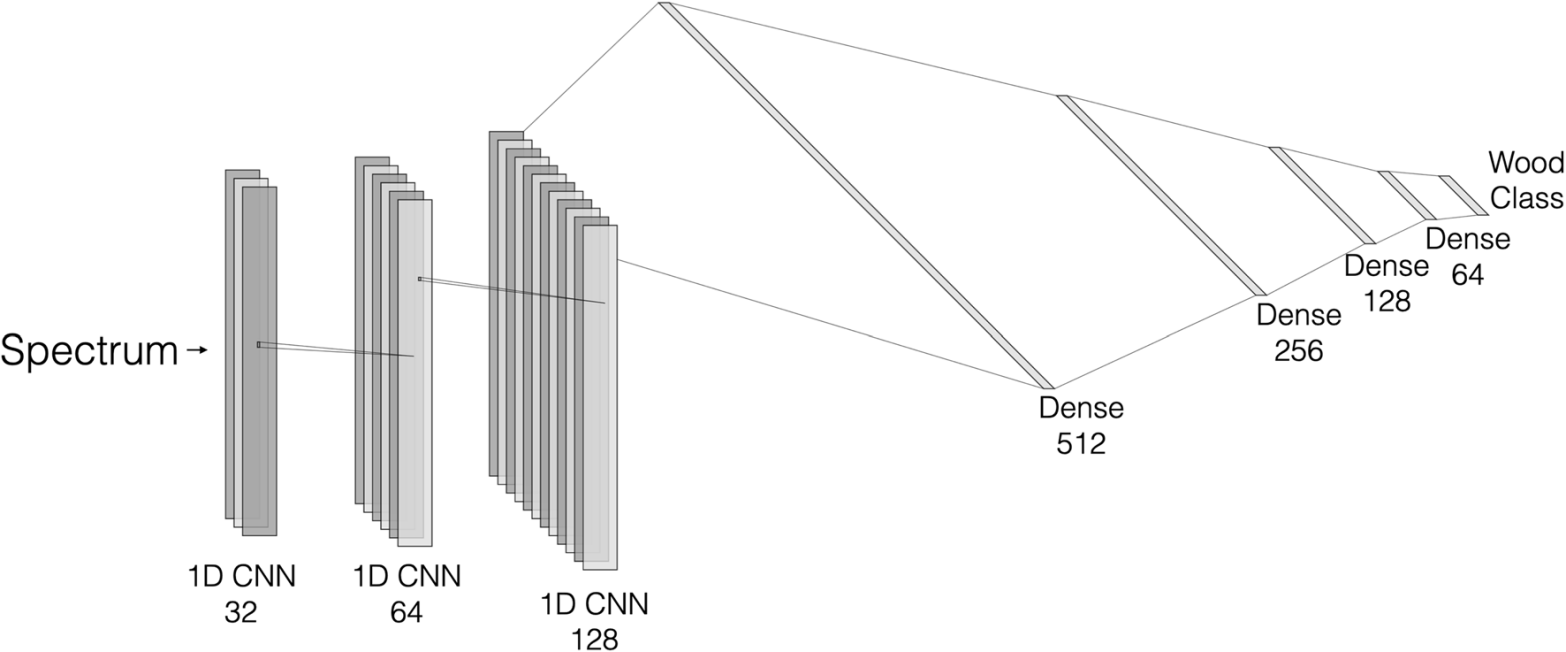
1D CNN Architecture for classification.

A one-dimensional convolutional neural network was generated using 3 1D CNN layers with filter sizes of 32, 64, and 128 with intermediate max pooling and batch normalization layers. Each 1D CNN layer had descending kernel sizes of 5, 3, and 2, which were fed into an interconnected network with 512, 256, 128, and 64 neurons with 60% dropout rates (Fig. 2).

This model was trained over 2000 epochs (e.g., forward and backward proportions of the network through the training dataset) using the Nadam optimizer, categorical crossentropy as a loss function, and a learning rate of 0.00001 with a batch size of 4. Fifteen percent of the data was randomly masked from training to be used for model validation.

A second smaller dataset including only wood samples with repeated representatives for each species was also used with the same model architecture and training method, though for only 1500 epochs. A third data set of random words with random spectra associations was made as a control to verify that the wood identification models were the product of meaningful signals, and not memorization by the network^36^.

The model is available in the open source SheetCrunch software^37^ using the Keras framework in Tensorflow 2.2.

## Results and discussion

Tree species identification was found to be 99% accurate in the cross validation set for all 48 species investigated.

**Figure 3:**
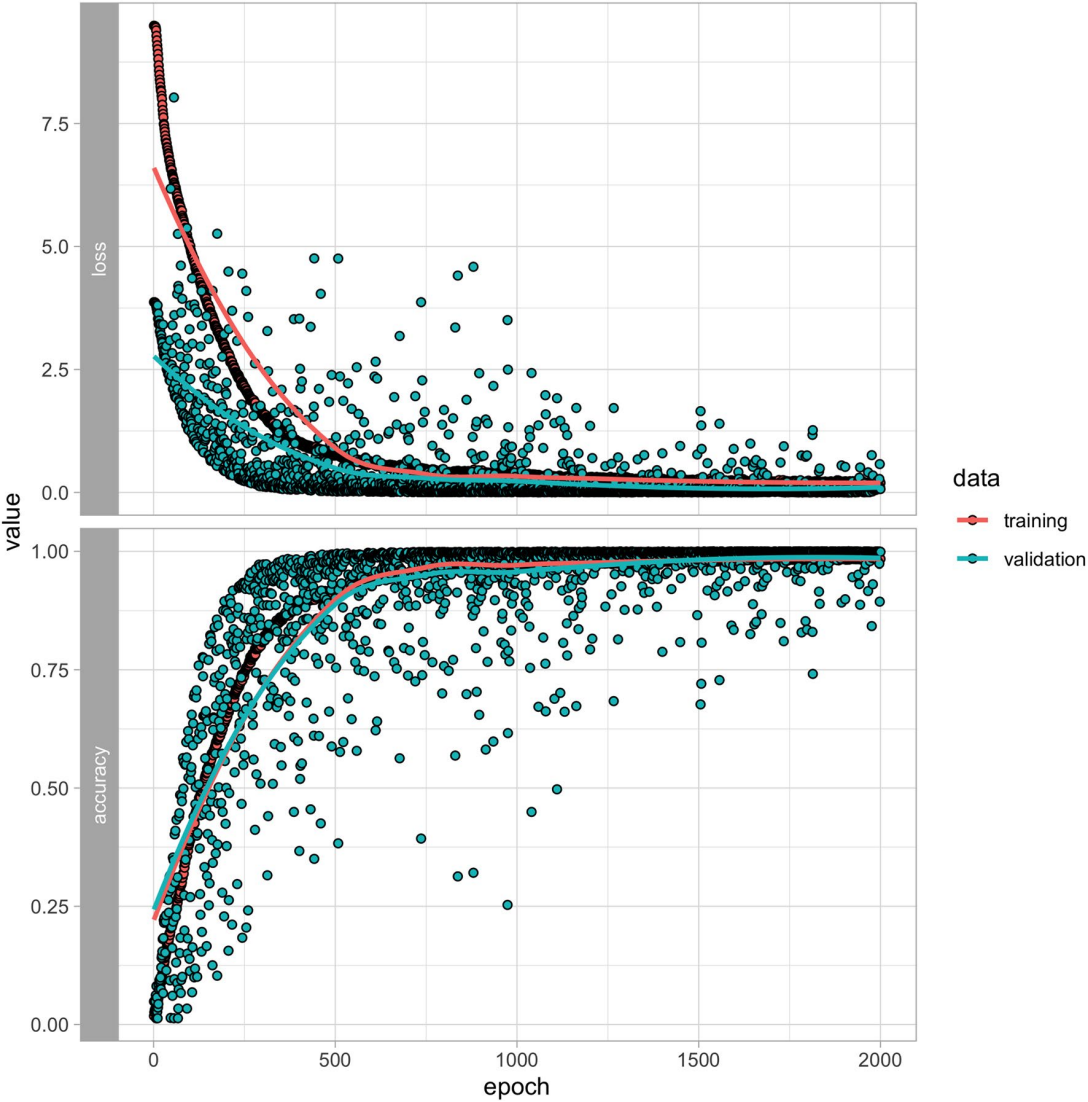
Training and validation loss (top) and accuracy (bottom) for the training (n = 14,672) and validation (n = 2590) portions of the full dataset. Models converged most of the time after 500 epochs, though considerable variation in validation accuracy is seen throughout training.

**Figure 4:**
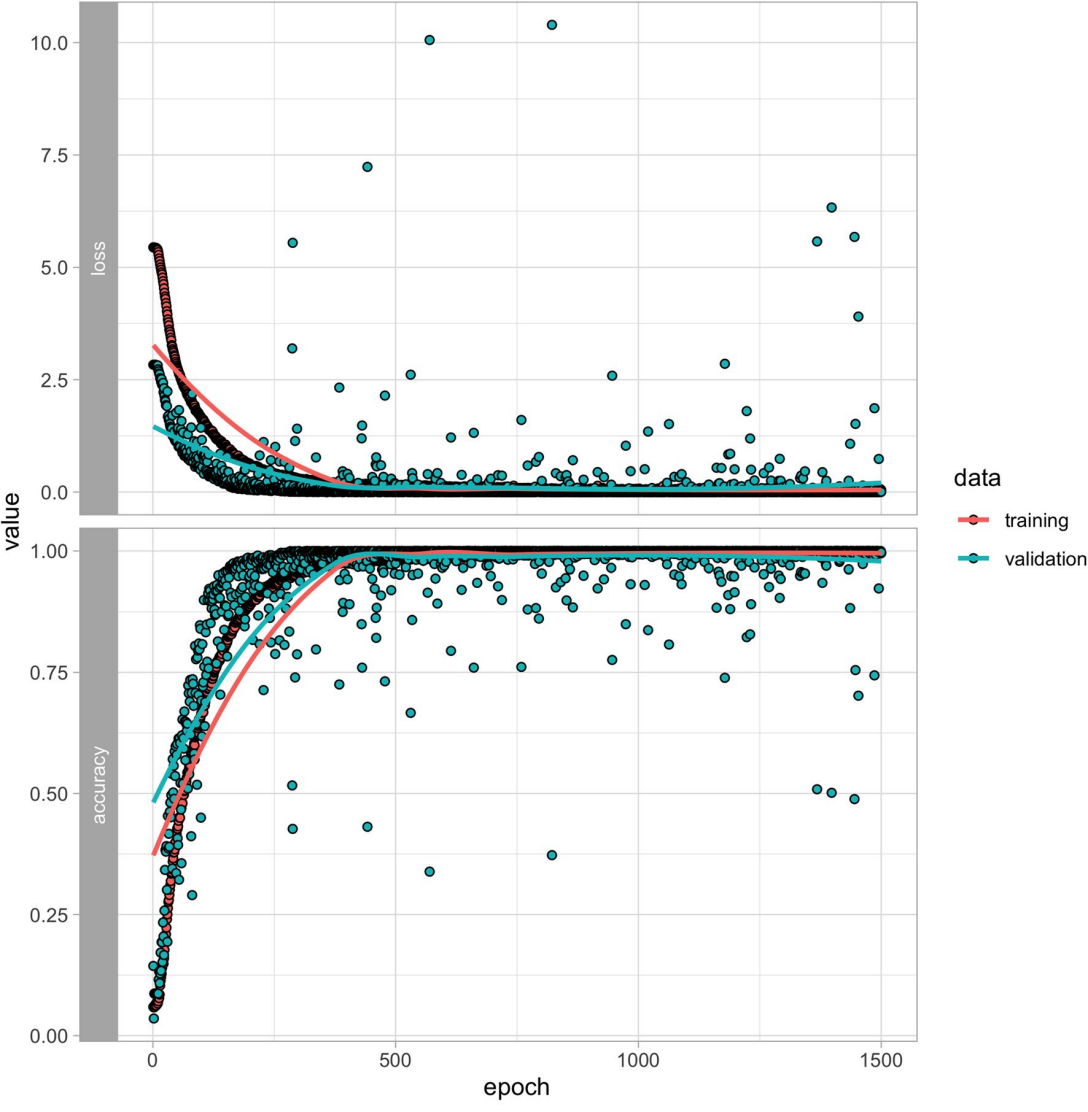
Training and validation loss (top) and accuracy (bottom) for the training (n = 7825) and validation (n = 1382) portions of the repeated dataset. Models converged most of the time after 250 epochs with a smaller range of validation accuracies compared to the full training set.

**Figure 5:**
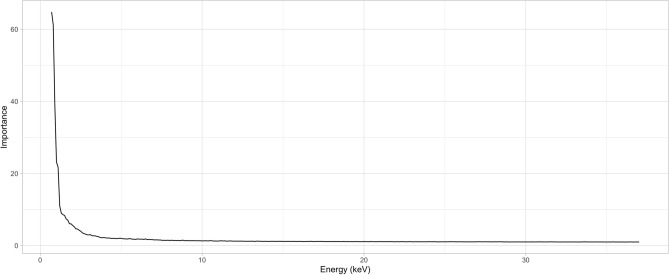
Importance features for the full dataset using categorical crossentropy as a loss function. Note that the most relevant information is included from 0.7 to 1.7 keV.

**Figure 6:**
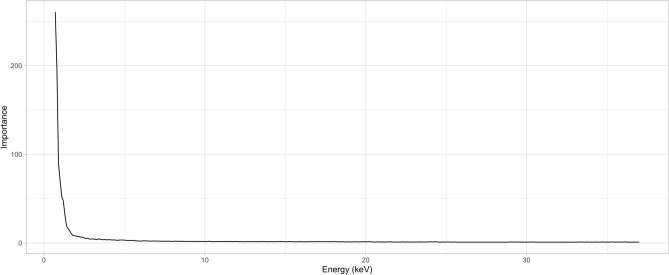
Importance features for the repeated dataset using categorical crossentropy as a loss function. Feature importance is largely identical to the full dataset.

The 1D CNN model (Fig. 3) converged after 500 epochs for the full data set and after only 250 for the partial data set (Fig. 4). Accuracy for the full model saw considerably higher variation in cross-validation results (Fig. 3, Supplemental Table 1) compared to the repeated model (Fig. 4, Supplemental Table 2), though both models have nearly identical feature importance plots (Figs. 5, 6). Training and validation accuracies were 98.5% and above (Table 2). Randomized data had much poorer performance with training and validation accuracies near 7% (Table 2, Supplemental Table 3).

**Table 2:** Final training and validation accuracy, sensitivity, specificity, and optimization precision for full, partial, and randomized data sets. Note that the high specificity for the random model is a product of it repeating the same prediction.

	N		Accuracy		Validation Mean Sensitivity (%)	Validation Mean Specificity (%)	Validation Mean Optimization Precision (%)
	Train	Validation	Train (%)	Validation (%)			
Full	14,672	2590	99.9	99.9	99.8	99.9	99.8
Repeats	7825	1382	99.9	99.7	99.7	99.8	99.6
Random	14,672	2590	7.2	6.9	3.4	96.7	− 93.1

The model performed well on wood identification for both the full and partial data sets (Table 2, see supplementary data for more detail). The accuracy was high enough to warrant concern that wood ID was not being addressed as much as the memorization of the data^33^. While randomized cross-validation in the manner presented in this manuscript is the traditional way of evaluating a model, an additional random word dataset with randomized associations to the spectra were generated as an additional test. If the same neural architecture could learn random associations as strongly as it learned wood taxa, then this could suggest either malfunctioning random cross-validation or data leakage (e.g., the validation data was included in the model training). The randomized data showed poor classification with training and validation accuracies near 7% (Table 2). For comparison, a fully random process would be approximately 3.5% accurate in identifying 29 classes. The higher performance in the random model was the result of uneven randomness in variables—it simply predicted one word (she) that had the most instances in the dataset. This is why the specificity of the random model is higher than would be expected (Table 2). The random model test illustrates that neither data memorization nor data leakage can explain the high accuracy in the wood taxon classification models.

Other machine learning approaches have inferred unusual properties from XRF spectra including water^20^ and carbon/nitrogen^38^ from organic fertilizers, organic carbon from sedimentary cores^21^, and meteorite classification^39^. While these approaches do not use 1D CNN models, they do highlight functions that are not typically considered for XRF. Machine learning expands the applicability of spectrometer-based technologies considerably beyond their traditional uses.

The full model saw accuracies as high as 100% and as low as 45% in the last 200 epochs (Fig. 3), while the range of validation accuracies is considerably narrower (Fig. 4). This is likely a product of having a single sample of a species represented for some groups. The multi-sample dataset provides a more representative example of how multiple observations of multiple objects is better suited to training networks.

Despite the limitation of single-sample beams to represent groups in the full dataset, the feature importance plots are nearly identical (Figs. 5, 6), with 0.7–1.7 keV being the most important portion of the spectra to classify wood. This is a surprising result as the elements that fluoresce in this region most prominently are sodium (Na), magnesium (Mg), aluminum (Al), and silicon (Si). Other elements found in woods include calcium (Ca), potassium (K), iron (Fe), sulfur (S), and phosphorous (P); however, all are found in rather small concentrations^40^. The majority of wood is comprised of carbon, oxygen, hydrogen, and nitrogen, mostly formed as cellulose and lignin. It is therefore unclear why this area of the spectrum is so important, but some hypotheses for the reason include:

1. The network infers most of what it needs to know from the first few keV of the spectra, making the rest of the spectrum redundant.
2. Variation in these elements may be more distinct (even in smaller concentrations) than previously thought, or components such as SiO_2,_ which is the main component of phytoliths, can be diagnostic for plant taxa.
3. The bremsstrahlung radiation reflected from 0.7 to 2 keV is reflective of density changes between wood taxa, which has been shown to be informative^18^.

A combination of these factors may well explain the nature of the model. Of the three hypotheses, number 1 is the most easily tested. By experimenting with different XRF spectra acquisition techniques (e.g., use an attenuating filter to minimize bremsstrahlung radiation) feature importance can be re-evaluated.

While this pilot study shows a high potential for success, there are some areas that need to be developed to corroborate the findings and create a more substantial working database. The samples investigated here derived from a study collection and may not meet the required taxonomic integrity of reference materials. Vouchered specimens should be analyzed to ensure that proper identification has been met^8^. Limited quantities of reference materials in existing collections and a lack of authenticated tree specimens worldwide^1,7^ make this challenging. The development of a comprehensive database is also required to advance XRF (or any other analytical technique) for the identification of tree species.

The samples investigated in this study were mostly heartwood, and the scans were mainly done on radial and tangential sections, or slight rotations between these two orientations. It would be necessary to test this system on sapwood and on transverse sections of wood to see if the same results followed suit, or if alternative calibrations would be required for these orientations. In addition, variation in moisture content was not considered for this study. Future work should consider investigating the effects of moisture content of the photons scattering.

Ideally with deep learning, the more data analyzed the better both across and within sample types. In this study, 48 tree species were tested with only 17 of those having multiple samples (mostly 2 samples and 3 samples for *Tilia americana*). This needs to be expanded to ensure that the findings for each of the tree species is indeed an identifier for that species and not more related to associated soil chemistry or geographical defining features (e.g., weather alteration may cause variation in density). In addition, looking at the same species with different known geographic locations would be necessary to assess the model’s validity for geographic variation in wood growth.

While deep learning traditionally requires significant hardware (typically GPUs), the 1D CNN model presented here can run on most CPU systems due to its lower dimensionality. Because the network is re-trainable (e.g., the neurons can be reloaded into a model), the data could be adapted for any XRF instrument provided its spectra are transformed in the same manner (0.7–37 keV in 100 eV increments), and there is some light retraining with representatives of the classes.

## Conclusion

This study presents the first use of XRF for the identification of tree species using a convolutional neural network machine learning model. This rapid, non-invasive, non-destructive analytical technique provides highly accurate identification of wood species. The findings show great promise for XRF’s use to help limit the illegal trade and transport of lumber. The ability to rapidly use XRF with limited training offers border guards the ability to make more accurate assessments of lumber and better triage which samples might require additional cross validation using more invasive techniques. Although this pilot study shows highly accurate identification, additional research should be undertaken to consider the effect and results of various wood orientations, moisture content, addition of biocides, and variations of species and geographic locations. A clear benefit to using non-invasive analytical techniques such as NIRS or XRF for wood analysis is that they can be used on finished products or timbers that are deemed too valuable from which to remove a sample. Rapid XRF species identification would also benefit other fields of study including the art market and art conservation, where it could aid in authentication and provenance studies as well as ensure the use of proper replacement components in conservation treatments.

## Supplementary Information

Supplementary Table 1.

Supplementary Table 2.

Supplementary Table 3.

Supplementary Legends.
