# Author Correction: Rapid identification of wood species using XRF and neural network machine learning

**DOI:** 10.1038/s41598-022-10650-w

**Published:** 2022-04-15

**Authors:** Aaron N. Shugar, B. Lee Drake, Greg Kelley

**Affiliations:** 1 Garman Art Conservation Department, Buffalo State College – SUNY, Buffalo, NY USA; 2 Department of Anthropology, University of New Mexico, Albuquerque, NM USA; 3 Collections, Curatorial and Conservation Branch, Indigenous Affairs and Cultural Heritage Directorate, Parks Canada, Government of Canada, Ottawa, ON Canada

Correction to: *Scientific Reports* 10.1038/s41598-021-96850-2, published online 02 September 2021

The original version of this Article contained an error.

In the Methodology section, under the subheading ‘Collection of spectra’,

“The scanning stage was set to move at 0.03 mm in the X axis per scan and then shift up 0.3 mm in the Y axis to create a matrix that produced approximately 260 scans per wood sample.”

now reads:

“The scanning stage was set to move at 3 mm in the X axis per scan and then shift up 3 mm in the Y axis to create a matrix that produced approximately 260 scans per wood sample.”

The original Article has been corrected.

