# Supplementary material for: Rapid identification of wood species using XRF and neural network machine learning
Source: Sci Rep. 2021 Sep 2;11:17533. doi: 10.1038/s41598-021-96850-2 (PMC8413463; doi:10.1038/s41598-021-96850-2)
Supplement: Supplementary file 4 — Supplementary Legends. [file 41598_2021_96850_MOESM4_ESM.docx]

**Supplementary Data Captions:**

**Table 1:** Taxon accuracy for train and test datasets for the full model.

**Table 2** Taxon accuracy for train and test datasets for the repeated model.

**Table 3:** Taxon accuracy for train and test datasets for the null randomized model.
